# Occipital Lobe Status Epilepticus, A Stroke Mimic with Novel Imaging Findings: A Case Report

**DOI:** 10.5811/cpcem.2022.1.55482

**Published:** 2022-07-26

**Authors:** Jordan Lawson, Wayne Triner, Brady Kluge

**Affiliations:** * Prisma Health Upstate, Department of Emergency Medicine, Greenville, South Carolina; † University of South Carolina, School of Medicine, Greenville, South Carolina

**Keywords:** case report, stroke mimics, hemianopsia, status epilepticus

## Abstract

**Introduction:**

Stroke mimics are a major diagnostic challenge during the initial evaluation of patients presenting with an acute focal neurological deficit. This case reviews a patient who presented to the emergency department (ED) with homonymous hemianopsia, a rare manifestation of focal status epilepticus of the occipital lobe. Her initial brain computed axial tomographic perfusion scan and magnetic resonance imaging revealed novel findings associated with this diagnosis.

**Case Report:**

A 70-year-old female presented to our ED with left visual field hemianopsia, dyskinesia, dysmetria, and facial droop. Her initial diagnosis was left posterior fossa circulation cerebrovascular accident. However, her neuroimaging indicated hypervascularity of the left occipital lobe without evidence of infarct or structural lesion. A cerebral angiogram excluded arteriovenous malformation. Subsequently, an electroencephalogram showed left occipital lobe status epilepticus.

**Conclusion:**

Hemianopsia is a rare presentation of focal status epilepticus mimicking stroke. Hypervascularity seen on advanced neuroimaging may have suggested this diagnosis on initial ED evaluation.

## INTRODUCTION

The initial emergency department (ED) diagnosis of ischemic stroke remains a clinical one. It is based upon the sudden loss of neurological function corresponding to a discrete vascular and anatomic distribution within the brain (and less commonly, the spinal cord). Generally, this diagnosis is accomplished by a focused history, which includes specific time of onset and subjective report of symptoms. This is coupled with an equally focused neurological examination designed to uncover a specific neurological deficit. The history and physical are augmented by laboratory and neuroimaging studies, which might reveal alternative explanations for the deficit such as hypoglycemia, and structural lesions within the brain. Additionally, neuroimaging is employed to exclude medical or interventional revascularization in the face of intracerebral hemorrhage.

The accuracy of stroke diagnosis has important implications since cerebral revascularization, whether systemic or interventional, carries risk of harm while incurring significant allocation of resources. Thus, stroke mimics, which account for up to 25% of admissions for probable strokes, are a major diagnostic challenge during the initial evaluation of patients presenting with an acute focal neurological deficit.^1^ Common stroke mimics include metabolic derangements, seizure, complex migraine, central nervous system infections, sepsis, non-stroke cardiovascular events, and functional disorders. To this end, methodologies, either already employed in early stroke evaluation or potentially available within the ED, may help physicians to identify uncommon stroke mimics with greater frequency. In this case report, we present a rare cause of a stroke mimic with novel neuroradiologic findings.

## CASE REPORT

A 70-year-old, functionally independent female presented to the ED by emergency medical services after her son had found her on the floor. She reported that she had “blurred vision.” The visual disturbance and fall were reported by the patient to have occurred 10 hours prior to ED presentation with associated left-sided “tingling and weakness.” She had a past medical history of hyperlipidemia, uncontrolled type II diabetes, carotid artery disease, coronary artery disease, stage III chronic kidney disease and hypothyroidism. She acknowledged poor adherence with her home medications, which included insulin lispro, insulin degludec, sacubitril/valsartan, torsemide, spironolactone, and metoprolol extended release.

On arrival to the ED, she had fluent speech, was managing her own airway, and had good peripheral perfusion. Her blood pressure was 131/61 millimeters mercury, heart rate 67 beats per minute, respiratory rate 18 breaths per minute, temperature 97.5° Fahrenheit, and oxygen saturation of 100% on room air. The initial evaluation was most notable for a left visual field hemianopsia. She also had left-sided dyskinesia and dysmetria with a subtle left facial droop. She had no other strength deficits. Her National Institutes of Health Stroke Scale score was 5 (age and month -1, mild left facial palsy – 1, hemianopsia – 2, limb ataxia – 1). The remainder of her physical examination was unremarkable.

Based on the focality of her neurological examination referable to a left posterior fossa lesion and being within the 24-hour window for mechanical clot extraction, our stroke team was mobilized. She underwent non-contrasted head computed axial tomography (CT), showing only mild atrophy. Being outside the window for systemic thrombolysis, she had a head and neck CT angiogram with perfusion study. This study revealed hyperperfusion of her left occipital lobe thought to be suggestive of an arteriovenous fistula (AVF). There were no apparent large vessel occlusions, infarcts, or penumbras. A brain magnetic resonance imaging (MRI) later that day offered similar findings without discrete evidence of an AVF.

Her initial laboratory studies in the ED included a white blood cell count of 11,800 cells per milliliter (mL) (reference range: 4000–11,500 cells/mL), hemoglobin of 14.5 grams per deciliter (g/dL) (12–18 g/dL), platelet count of 446 platelets/mL (125–415 platelets/mL). She had a serum sodium of 131 millimoles per liter (mmol/L) (133–144 mmol/L), potassium of 4.5 mmol/L (3.4–5.1 mmol/L), chloride 91 mmol/L (101=111 mmol/L), bicarbonate ion of 27 mmol/L (20–30 mmol/L), a calculated glomerular filtration rate of 40 mL per minute (min) (reference: greater than 59 mL/min), and an elevated serum glucose of 583 milligrams (mg)/dL (82–99 mg/dL). Her urine toxicology screen did not show the presence of amphetamines, benzodiazepines, cannabinoids, cocaine, or opioids. Her electrocardiogram revealed non-specific ST-segment abnormalities.

**CPC-EM Capsule**

What do we already know about this clinical entity?

*Stroke mimics are common and important to identify. However, findings of stroke mimics can be subtle*.

What makes this presentation of disease reportable?

*This is a report of a stroke mimic, occipital lobe status epilepticus, previously undefined in the emergency medicine literature, with novel findings on imaging*.

What is the major learning point?

*Perfusion studies carried out during an acute stroke evaluation may lead the clinician to consider this and other presentations of complex status epilepticus*.

How might this improve emergency medicine practice?

*Understanding this finding on imaging may lead the physician to consider the diagnosis of stroke mimic and redirect care appropriately*.

Following initial stroke assessment, our patient was then admitted to the hospitalist service for in-patient evaluation of her unexplained neurological deficits and continued concern for an AVF. On hospital day two, due to persistence of the unexplained neurologic deficits the patient underwent a bedside electroencephalogram (EEG). This study revealed left occipital lobe focal status epilepticus. This prompted administration of levetiracetam 1500 mg loading dose intravenously and 750 mg orally twice daily. The following day, her left visual field deficit persisted. On day three of hospitalization, a cerebral angiogram excluded an arteriovenous malformation and revealed only several non-critical stenoses. A long-term EEG showed persistent left occipital lobe status epilepticus prompting the addition of lacosamide 200 mg twice daily during her hospitalization. She was then transferred to an in-patient rehabilitation facility for mobility and muscle conditioning.

Seven days following her initial presentation, physical medicine and rehabilitation documented that she had regained full visual fields with no dyskinesia or dysmetria. Unfortunately, 30 days following initial presentation, our patient was re-admitted to our hospital with acute cholecystitis. During that hospitalization, she had a downward course that included a large cerebral infarct culminating in withdrawal of support.

## DISCUSSION

Our patient had several stroke risk factors including advanced age, diabetes mellitus, hyperlipidemia, and renal dysfunction. Her initial evaluation was dominated by a dense left hemianopsia and left-sided dysmetria and dyskinesia, thus localizing her deficit to her left posterior fossa. Our initial evaluation pointed us toward ischemic stroke, since hemianopsia can occur in as many as 7% of all strokes.^2^ It is possible her seizure may have been associated with a transient ischemic attack (TIA). This, however, did not negate the importance of her seizure, since that was the diagnosis requiring expeditious treatment.

The incidence of focal occipital status epilepticus is rare and unknown other than found in a handful of case reports.^3^,^4^,^5^,^6^ Occipital lobe status epilepticus may present in several fashions. There may be “negative” features such as unilateral vision loss, as our patient presented. This should not be confused with a structural cause of vision loss (tumor) and secondary seizure. Likewise, there may be “positive” features such as visual hallucinations or flashing lights, which may be difficult to distinguish from occipital migraine, but for EEG findings of epileptiform activity. It is possible for propagation of the seizure to include adjacent areas of the brain to incorporate oculomotor or other motor features.^7^ To our knowledge, this is the first occurrence of occipital lobe status epilepticus to be reported in the emergency medicine (EM) literature.

Hyperperfusion of the left occipital lobe seen on perfusion CT angiography and MRI, may represent preliminary findings associated with focal status epilepticus.^7^,^8^ This is the first report of this finding to our knowledge to be reported in the EM literature. While this finding would be more likely associated with an AVF, one was not visualized by angiography or MRI. An EEG subsequently revealed her underlying diagnosis: focal status epilepticus of the left occipital lobe.

Our patient had a well-defined focal neurological deficit, which after initial imaging went unexplained. This resulted in a 48-hour delay in diagnosis and treatment. This could have been averted had focal status epilepticus been considered. Should this diagnosis have entered consideration earlier, emergency EEG acquisition could have been obtained in the ED through either conventional or point-of-care EEG monitoring.^9^,^10^

## CONCLUSION

Two features of this case have importance for emergency physicians. The first is the finding of hemianopsia as a stroke mimic resulting from focal status epilepticus. This presentation of stroke mimic appears to be very rare and has not previously been reported in the EM literature. This contrasts with the relatively common occurrence of hemianopsia resulting from ischemic stroke. Second, cerebral hypervascularity implied by both CT angiography and MRI may be a finding associated with focal status epilepticus. This association has recently gained attention in the neuroradiology literature.^6^, ^7^, ^8^ Hyperperfusion, however, is not limited to seizure. It has been demonstrated following reperfusion of cerebral vessels as might occur with TIA or therapeutic reperfusion.^11^

In the context of contemporary stroke care, CT perfusion of the brain is now commonly carried out in centers with access to invasive neuroradiology to determine the appropriateness of invasive neuroradiology in those patients for whom systemic thrombolysis is contraindicated. This finding may then be reported to the emergency physician who will need to assess its clinical relevance. Yet the performance characteristics of this finding (sensitivity and specificity) are still undefined. In conclusion, hemianopia although a rare manifestation of focal status epilepticus merits consideration as a possible stroke mimic and may also carry unique radiologic findings.

## References

[1] Gibson LM, Whiteley W (2013). The differential diagnosis of suspected stroke: a systematic review. J R Coll Physicians Edinb.

[2] Zhang X, Kedar S, Lynn MJ (2006). Homonymous hemianopia in stroke. J Neuroophthalmol.

[3] Siatouni A, Gatzonis S, Alexopoulos A (2016). Simple partial status epilepticus manifested as homonymous hemianopsia: a rare intracranial recording. Clin Pract.

[4] Yang Y, Zhang S, Duan J (2020). Acute visual impairment as a main presenting symptom of non-convulsive status epilepticus: a case report. BMC Neurol.

[5] Matsumoto Y, Akamatsu Y, Ogasawara Y (2020). A case of paroxysmal homonymous hemianopsia: uncommon presentation of nonconvulsive status epilepticus. Radiol Case Rep.

[6] González-Cuevas M, Coscojuela P, Santamarina E (2019). Usefulness of brain perfusion CT in focal-onset status epilepticus. Epilepsia.

[7] Schäffler L, Karbowski K (1988). Zur Frage der epileptischen Aktivität des Okzipitallappens. Klinisch-elektroenzephalographischer Beitrag. Fortschr Neurol Psychiatr.

[8] Shimogawa T, Morioka T, Sayama T (2017). The initial use of arterial spin labeling perfusion and diffusion-weighted magnetic resonance images in the diagnosis of nonconvulsive partial status epileptics. Epilepsy Res.

[9] Gugger JJ, Llinas RH, Kaplan PW (2020). The role of CT perfusion in the evaluation of seizures, the post-ictal state, and status epilepticus. Epilepsy Res.

[10] Rittenberger JC, Weissman A, Baldwin M (2019). Pittsburgh Post Cardiac Arrest Service. Preliminary experience with point-of-care EEG in post-cardiac arrest patients. Resuscitation.

[11] Brenner JM, Kent P, Wojcik SM (2015). Rapid diagnosis of nonconvulsive status epilepticus using reduced-lead electroencephalography. West J Emerg Med.

[12] Copen WA, Schaefer PW, Wu O (2011). MR perfusion imaging in acute ischemic stroke. Neuroimaging Clin N Am.

